# Total Worker Health: A Small Business Leader Perspective

**DOI:** 10.3390/ijerph15112416

**Published:** 2018-10-31

**Authors:** Janalee Thompson, Natalie V. Schwatka, Liliana Tenney, Lee S. Newman

**Affiliations:** 1 Center for Health, Work & Environment, Colorado School of Public Health, University of Colorado, Anschutz Medical Campus, 13001 E. 17th Pl., 3rd Floor, Mail Stop B119 HSC, Aurora, CO 80045, USA; janaleethompson7@gmail.com (J.T.); liliana.tenney@ucdenver.edu (L.T.); lee.newman@ucdenver.edu (L.S.N.); 2 Department of Environmental and Occupational Health, Colorado School of Public Health, University of Colorado, Anschutz Medical Campus, 13001 E. 17th Pl., 3rd Floor, Mail Stop B119 HSC, Aurora, CO 80045, USA; 3 Department of Epidemiology, Colorado School of Public Health, University of Colorado, Anschutz Medical Campus, 13001 E. 17th Pl., 3rd Floor, Mail Stop B119 HSC, Aurora, CO 80045, USA; 4 Division of Pulmonary Science and Critical Care Medicine, Department of Medicine, School of Medicine, University of Colorado, Anschutz Medical Campus, 13001 E 17th Pl., Aurora, CO 80045, USA

**Keywords:** workplace safety, safety leadership, health promoting leadership, safety programs, health promotion, health protection, leadership, qualitative study

## Abstract

Total Worker Health^®^ (TWH) frameworks call for attention to organizational leadership in the implementation and effectiveness of TWH approaches. It is especially important to study this within in the small business environment where employees face significant health, safety, and well-being concerns and employers face barriers to addressing these concerns. The purpose of this study was to gain a better understanding of how small business leaders perceive employee health, safety, and well-being in the context of their own actions. We conducted semi-structured interviews with 18 small business senior leaders and used a qualitative coding approach to analyze the transcripts to determine the frequency with which leaders discussed each code. When we asked leaders about their leadership practices for health, safety, and well-being, leaders reflected upon their business (65%), themselves (28%), and their employees (7%). Leaders rarely discussed the ways in which they integrate health, safety, and well-being. The interviews demonstrate that small business leaders care about the health of their employees, but because of the perceived value to their business, not to employees or themselves. Thus, they may lack the knowledge and skills to be successful TWH leaders. The present study supports a need for continued small business TWH leadership research.

## 1. Introduction

Total Worker Health^®^ (TWH) is defined as policies, programs, and practice that integrate protection from work-related safety and health hazards with promotion of injury and illness prevention efforts to advance worker well-being [1]. As the field of TWH gains research and practice support, it is important to study the role of organizational leadership. Several TWH frameworks call for attention to organizational leadership in the implementation and effectiveness of TWH approaches [2,3,4]. However, to date, leadership research has rarely integrated both health promoting leadership and safety leadership. Doing so can improve our understanding of how to assess and improve TWH-specific leadership practices to ensure TWH system effectiveness [5]. This is especially important in the small business environment where employees face significant health, safety, and well-being concerns [6,7] and employers face barriers to addressing these concerns (e.g., resources) [6,8]. Therefore, as a first step, the purpose of this study was to improve our understanding of how small business leaders perceive employee health, safety, and well-being in the context of their own actions. 

The field of leadership research has grown substantially over the past few decades. Dinh et al. [9] found 66 leadership theory domains in their review of the leadership literature. While the evidence base for each of these theories varies, it is generally agreed that leadership is a significant contributor to organizational culture and ultimately, organizational success [10]. Successful organizations primarily have leaders who adopt several leadership characteristics to best meet organizational needs [11]. A healthy business culture is derived from integrating leadership theories that align employee and organizational goals [12], though some leaders who use some styles of leadership have been more effective in promoting health than others.

Building positive relationships, empowerment and the ability to view the organization from an employee viewpoint are characteristics of successful leaders. Servant leaders are known for their supportive nature and how they achieve organizational goals by prioritizing needs of employees first [13]. Similarly, leaders who employ high quality relationships (leader-member exchange (LMX)) leverage employee relationships to meet organizational ambitions [14]. Successful leaders also place value in employee perceptions of meaningful work and contribution to the larger picture. Sirota’s Three Factor Theory argues for building and maintaining employee enthusiasm through equal treatment, employee belief that work is meaningful, and camaraderie. Sirota’s theory describes that when leaders champion these factors, workers will be enthusiastic and motivated to produce more, while enjoying what they do [15].

Leadership has an impact on employee physical and psychological health. Transformational leadership, specifically, has been positively associated with psychological well-being [16], stress [17], depression [18], and sleep quality [19]. In terms of safety-specific outcomes, several meta-analyses demonstrate that leadership (generally defined) and transformational leadership are related to better safety climate, better safety practices, and fewer occupational injuries [20,21,22]. However, employee health can also be compromised as a result of poor leadership. Researchers have found associations between poor management support and ischemic heart disease [23], elevated blood pressure [24], problem drinking [25], smoking [26], as well as mental health issues related to affective well-being [27] and job well-being [16,28,29]. For example, Skakon et al. found that when leaders have high stress and poor affective well-being, their subordinates also have high stress levels and poor well-being [27].

Recently, researchers have begun to investigate health promoting leadership. Health promoting leadership theory involves leadership characteristics that encourage wellness at work [30]. Research has shown that leaders who promote workplace health are hands-on, supportive [31], demonstrate health awareness, and value community and fairness [32,33]. It has also been suggested that work-related stress may be reduced when specific, health promoting transformational leadership skills are demonstrated at work [34]. Though research may be limited, all health promoting leadership studies suggest that health promoting leadership characteristics are likely to produce positive health outcomes. However, in the context of TWH, health promoting leadership fails to consider the role of safety leadership.

In contrast, the theory and evidence base for safety leadership gained traction over fifteen years ago [35]. The majority of the safety-specific literature focuses on Bass’s multifactor leadership theory [36]. Some researchers developed safety-specific assessments of transformational and passive leadership and found them to be more associated with safety outcomes than general forms of leadership [37]. Studies have shown that the passive form of transactional safety leadership negatively impacts safety outcomes (e.g., injury rates) whereas transformational safety leadership positively impacts safety outcomes [38]. However, other more active forms of transactional leadership, contingent reward and active management-by-exception, have been linked to positive safety outcomes [39]. Safety leadership intervention studies have suggested that training leaders on transformational safety leadership skills can lead to positive safety outcomes [40,41]. Other researchers applied the LMX theory [42] and empowering leadership theory [43] to workplace safety and found both to be related to positive safety outcomes. Although, similar to the singular focus of HP leadership, safety leadership research has focused solely on safety and has rarely included research on other outcomes. 

The literature illustrates the segmented nature of leadership research in the employee health promotion and safety contexts. Health promoting and safety leadership are both “best practices” to support worker health and safety but we are unaware of a concerted effort to evaluate their use in synchrony. Furthermore, we are aware of only one study that assessed health promotion and safety leadership support amongst small businesses. They found that small business leaders who advocate for TWH have higher integration scores than leaders who do not [44]. However, this study did not define TWH leadership support or describe the ways in which TWH leaders advocate. Thus, while there is some indication that TWH leadership is important amongst small businesses, it is unclear how small businesses leaders can demonstrate support for TWH. For this reason, as a first step in understanding TWH leadership development needs, we sought to interview small business leaders to understand how they discuss their use of practices that demonstrate a commitment to health, safety, and well-being.

## 2. Materials and Methods

We conducted a qualitative study with small business leaders to understand their current approach to leadership for employee health, safety, and well-being (Appendix A). We recruited small business leaders (<500 employees) in Colorado and Wyoming from a variety of industries by phone and email from May 2017 to August 2017. Businesses were identified through existing networks including a workers’ compensation insurer, chambers of commerce, and a community-based program, Health Links™. Leaders were eligible to participate if they had significant decision-making power in the organization (e.g., owner, senior executive, or CEO).

The first author conducted 30-min, semi-structured interviews in-person or by phone asking each leader how they perceive health, safety, and well-being in their organization and their role in shaping it. The authors generated eleven interview questions based on the following themes related to key theories used in previous health promotion or safety leadership research: organizational mission, organizational culture, leading by example as well as employee advancement, feedback, and recognition [11,15,37,45,46,47]. We chose these theories because they reflect important leader characteristics and actions for employee health, safety, and well-being. Beyond the transformational style commonly studied in safety leadership [11,37], other styles layer in important leadership aspects, including openness, ethics [45], equity [15], focus on follower needs [47], and relationships [46]. In keeping with the TWH framework, all questions inquired about leader actions associated with health, safety and well-being together. In keeping with leadership frameworks, all questions asked them about their own practices, not their general business practices. If the interview lasted less than 30 min, the first author asked additional follow-up questions. Follow-up questions were developed a priori, however they were chosen by the interviewer based on participant answers to main survey items. The interview questions can be read in the supplementary material. All interviews were recorded. We obtained verbal permission from all participants prior to recording. The first author transcribed the interview by hand in Microsoft Word and then imported it into Dedoose, qualitative data analysis software [48]. Our protocol and all study procedures were approved by the Colorado Multiple Institutional Review Board.

### Analysis

The first and second authors analyzed the interview data using a qualitative coding approach described by Saldana [49]. We used descriptive codes to summarize content line by line into concise themes. The authors initially coded two transcripts individually with codes that best represented the interview content, allowing the codes to emerge from the content. The authors then met to compare codes and determine which codes best represented the content. Manuscripts were coded two at a time until all coding was complete. During this initial coding phase, three overarching themes became apparent. Thus, the authors decided to categorize all codes into one of three overarching themes. First, a *business* overarching theme included transactional policies, programs, and practices. To be coded within this overarching theme, the leader must have spoken about how their business operates in general and not specific to either what they do or say or what their employees do or say in a health and safety context. Second, an *employee* overarching theme represented a discussion of health, safety, and well-being from their employee’s perspective. Finally, a *self* overarching theme represented a discussion of what they personally do for health, safety, and well-being.

The pair met after initial coding to compare and discuss their respective coding scheme. Codes were structured as: parent code (three overarching themes), child code (sub-code), grandchild code (sub-sub-code). It was agreed upon a priori that lines of code could be simultaneously coded as more than one code, if deemed necessary. When codes differed between investigators, a discussion took place and the most agreeable code was assigned. After each combined coding session, we transferred the finalized codes into Dedoose [48]. This process was followed until all transcripts were coded. Once the coding phase was completed, we extracted the coded data from Dedoose to Microsoft Excel. Finally, we conducted a descriptive, quantitative analysis of the overall frequency of codes.

## 3. Results

**Figure 1:**
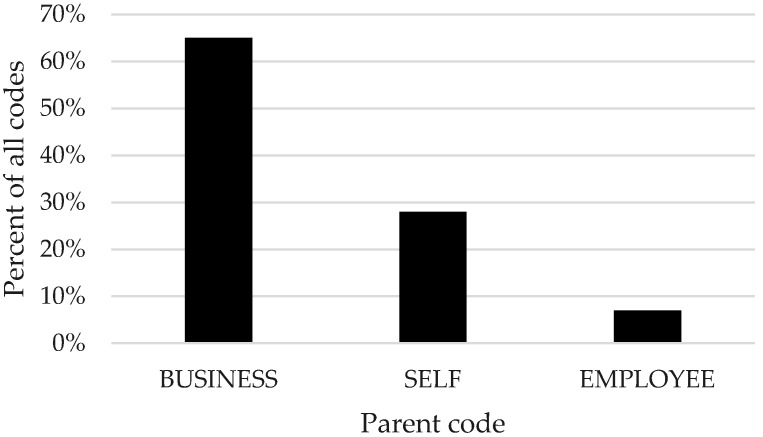
Percent of total codes mentioned by parent code.

In total, we interviewed 18 small business leaders from diverse industries based on the 10 Occupational Safety and Health Administration (OSHA) industry divisions. Industries were represented as follows: (A). Agriculture, Forestry, Fishing, 4; (B). Mining, 1; (C). Construction, 2; (D). Manufacturing, 1; (E). Transportation, Communications, Electric, Gas, Sanitary Services, 2; (G). Retail trade, 1; (H). Finance, Insurance, Real Estate, 1; and (I). Services, 6. About half of the leaders were female (n = 8.44%). Overall, the most common overarching theme discussed was their business, which represented 66% of total codes. Leaders discussed themselves and their employees much less frequently (see Figure 1). In the following sections, we describe each of these three overarching themes by highlighting the top five child codes within each theme. There were no qualitative differences in responses by the gender of the leader.

### 3.1. Business

Small business leaders primarily talked about health, safety and well-being in the context of the business they owned or worked for. The most frequent child codes in a business context, in order from most to least frequently mentioned, included health and safety programs, organizational barriers to doing TWH, gathering employee feedback on health and safety, communicating the importance of health, and program evaluation (see Table 1).

Within this overarching theme, health and safety programs were overwhelmingly referenced the most with leaders mentioning a variety of program elements. The most common element was employee incentives. Most small business leaders either discussed the current use of incentives, or desire to implement incentives to increase employee participation in health and wellness activities.

“We tied it to an incentive program. So we pay out incentives three times a year… we are one of the few remaining family-owned businesses that still pay full benefits for our 30 management team members and their entire family…if someone can’t participate in one of the platforms that we choose and they can’t take and invest that time for themselves and go get a health assessment and be active, that maybe they don’t get an incentive”.

“For participating in a lot of these activities, just by signing up to do yoga or signing up to give blood, we do random drawings for gift cards… After the event, we publicize how many people participated and who got the gift card”.

Other common, but less frequently mentioned, grand-child codes under health and safety programs included safety and wellness meetings and/or committees as well as health insurance offerings, such as benefits and biometric screenings. Compared to larger businesses, it is not as common for small businesses to offer health insurance benefits to employees, however, a few of the leaders in our study did mention offering these benefits to their white-collar workforce (see quote above under incentives).

“We also conduct monthly safety meetings in the field and all of the employees in the field attend. We do keep attendance at it and we’ve begun to integrate a variety of things. [There is an] enhanced level awareness and knowledge base at those meetings”.

“Through our benefits program…once a year or twice a year we have the third party come in and do the health assessments where they do the blood pressure, cholesterol levels and readings and it’s interesting because you can see the history through the years…it’s just interesting just to see how that changes throughout a period of time”.

“We started to, about a year and a half ago, provide some of our more management level positions and some of our full-time employees with health insurance”.

It is worth noting that leaders rarely discussed health, safety, and well-being practices from an integrated perspective. Leaders mentioned a variety of program elements from paid time off to job hazard analyses processes, but only one leader mentioned that they took steps to integrate these efforts. Another leader recognized the need for their business to integrate their efforts to protect and promote employee health. However, it was somewhat common for leaders to compare their safety and health promotion programs. About one-third of the leaders mentioned that one program was in better shape than the other.

“We definitely separate health and safety. When we think about safety, we think about workplace accidents only. We don’t connect the two, so I think one of the future things I would like to do is find an overlap of them”.

Within this overarching theme, leaders also discussed organizational barriers associated with doing TWH. Leaders mentioned barriers such as an offsite workforce (e.g., easier to give office employees resources than field workers), difficulty obtaining employee engagement (e.g., challenge of designing programs that reach employees), and generational differences (e.g., millennials being perceived as unreliable and not working as hard as older generations). However, if they are able to successfully implement programs, some leaders (7% of all business child codes) mentioned some perceived beneficial business outcomes of adopting TWH, including better employee health and improved employee engagement. 

Leaders also discussed the ways in which their business communicates health information to their employees and gathers employee feedback on health and safety programs. When discussing health communication, leaders frequently mentioned different modes of communication such as email, newsletters, and postings on wall boards. When discussing the ways in which their business goes about gathering feedback, leaders mentioned surveys, annual performance reviews, direct communication between the safety/wellness committee and employees, near miss injury reporting programs, etc. Some leaders also discussed efforts to evaluate their programs and take action to improve their programs, for example, not only asking for employee feedback, but also taking steps to act upon that feedback.

“Then we have a newsletter that goes out to all of our employees and again, there is something in there about safety, something in there about your health and other things”.

“Information that we get through the near-miss program can also show us what employees are looking for and what they’re needing and just kind of assessing that and prioritizing that and seeing if there’s something that we can do about it”.

### 3.2. Self

About one-third of the time, leaders spoke about workplace health, safety, and well-being from their own perspective. In order from most to least frequently mentioned, included leading by example, demonstrating individual consideration, helpful strategies for engaging employees, outcomes they perceive by personally engaging in TWH-focused leadership practices, and value for health. Leading by example was most commonly expressed as the leader participating in health and wellness initiatives or activities. Notably, “participate” was a grandchild code of “lead by example” and represented 15% of the “lead by example” codes. Individual consideration was denoted when a leader gave an example of how they paid special attention to individual employee to check-in or to help them. Some leaders specifically mentioned having an open-door policy to provide a safe place for employees to talk about both work and non-related work issues. Others also stressed the importance of connecting with employees on an individual level.

“You can’t expect people to pull away from their desks and come participate in something if it’s not important enough for you to do it yourself”.

“I hope that my actions positively affect the behaviors of my employees…if I come into the office in a bad mood, others are also in a bad mood. If I come into the office psyched and engaged, others are psyched and engaged. As a leader, I get that people mirror and model my behavior”.

“One thing that I tried to do over the years able to listen to my coworkers and just, you know be willing to hear them and how they’re doing and be able to help in any way that I can”.

“…we have an open-door policy… if you’re struggling whether or not it’s work-related or outside of work… you know we are here for you. We get life…we [upper management] tried to take our own personal experiences and reach out with regards to how we can involve our employees”.

“But there are tremendous opportunities when you do connect with someone… they know you understand what they’re doing and that you have their best interest in mind. You can make a really meaningful change by simply helping someone do something the right way so they don’t get hurt, or helping them achieve a professional development goal that they’ve always wanted to do, whether it’s a training, or a certification…”

Of the few times leaders mentioned their health values, they spoke from a couple of perspectives. For example, some mentioned it terms of a personal value while others mentioned it in relation to their work team.

“And another thing I’d add is that I truly care about the well-being of the people that I work with and that are my co-workers. From a human perspective, it just matters to me that they feel as good as they can”.

“I think it’s important from a personal perspective not only for ourselves, but also for our employees and that they conduct themselves in a safe manner so that they return to their families safely and be healthy and be able to provide for their families”.

On several occasions, leaders mentioned strategies they found to be helpful as they personally worked to promote employee health, safety, and well-being.

“First and foremost, it’s building a trust level”.

“We have an advantage because we are small, and I think it’s critical in a small business environment that you need to develop a structure that tunes it for each individual employee”.

When leaders spoke about perceived outcomes of their personal efforts to engage in TWH, they most commonly referred to better employee/leader relationship and employee engagement.

“I think that I have so many people that want to work with us and with me, and with my partners just because they see us doing what we’re doing”.

### 3.3. Employee

In order from most to least frequently mentioned, leaders mentioned employees as a barrier to program effectiveness, employees who are treated like family, employees demonstrating leadership in the program, employees participating in workplace health and safety programs, and individual accountability. Leaders mentioned difficulty in getting employees engaged in the programs for several reasons, such as adding more work to their busy schedules and cultural differences.

“The reality is that everyone is juggling things like having a family, getting their work done…having a hard time finding time to fit it exercise or gardening or all of the great stuff that we’d do if we only worked 30-h weeks”.

“It matters from an employee perspective obviously because we have got a small workforce… We wear a lot of different hats”.

“Our employee base is mostly Latino based and in most cases there is a difference of cultures between their originating country and here in the United States”.

Business leaders also expressed an understanding that employees have other non-work responsibilities, like family. Some even promoted family inclusion to increase participation in health and wellness initiatives.

“…people can bring their families and their pets”.

Some leaders mentioned that their employees demonstrate leadership. For example, leaders mentioned instances in which employees identify hazards and help to control them, help build safety and/or wellness programs, and senior employees who voluntarily coach newer employees until they become familiar with safe work processes.

“I think it’s important to involve employees in that decision-making process and development of programs”.

“So we take somebody who is extremely knowledgeable and skilled and they have to oversee someone actually executing on a procedure before they sign off that they are competent to do that”.

Finally, as it pertains to health in general, some leaders mentioned that ultimately employees have to make the decision to be healthy.

“We just try to leave it up to the individual to make their own decisions about what they are comfortable with”.

## 4. Discussion

We sought to understand TWH from the small business leader perspective. When we asked leaders about their leadership practices for health, safety, and well-being, leaders reflected upon their business 65% of the time, on themselves 28% of the time, and their employees 7% of the time. These findings demonstrate that small business leaders primarily communicate about TWH through the lens of their business policies and programs. Within each of these three overarching themes, leaders most commonly discussed elements of their TWH programs followed by the ways in which they lead by example within these programs and the employee barriers to TWH program effectiveness. Leaders rarely discussed the ways in which they integrate health, safety, and well-being.

Historically, leadership research focused on individual leader qualities and practices. The leaders in our study mentioned several times that they lead by example and considered the individual needs of their workforce, both of which were previously linked to health and safety outcomes [39]. However, other positive leadership characteristics such as empowering employees, coaching and teaching, motivating and inspiring, being authentic and ethical, and sharing or distributing leadership were rarely mentioned in the interviews [36,45,47,50,51]. Leaders mentioned business practices to encourage employee growth (i.e., training), solicit employee feedback, and communicate health messages. However, they did not specifically mention the ways in which they personally engaged in these practices.

Present day leadership research expands the concept of leadership from the individual leader to followers, the work environment, culture, etc. [52]. Our finding that small business leaders overwhelmingly discuss health, safety and well-being in the context of their business when asked about TWH leadership practices highlights the importance of considering leadership in a more comprehensive context. Similar to other qualitative studies on management perceptions of safety or health promotion programs [53,54], leaders mentioned a variety of policies and programs they have for their employees, as well as barriers to program effectiveness and outcomes of the program if successful. This perspective focuses on what Burke et al. [55] would call a transactional rather than transformational perspective. A transactional perspective focuses on management issues (e.g., TWH structures and systems), whereas a transformational perspective focuses on leadership, culture, and overall organizational mission and vision. Both are important aspects of a business’s TWH strategy [7]. Our findings point to an opportunity to study leadership practices in the context of existing TWH systems, especially as it pertains to integration. It also demonstrates a need to help small business leaders connect their own practices to their business’s TWH structures and systems in practice [52]. 

In our study, leaders rarely talked about their employees’ perspective of health, safety, and well-being. When leaders did, they talked about employees in the context of barriers to program effectiveness. Small businesses leaders who wish to increase employee engagement must be able to understand and describe their employee’s perceptions of the TWH program. Safety climate research demonstrates the importance of employee perception, showing that employees often report worse perceptions of organizational or management commitment to safety than managers [56,57]. Huang et al. [56] argues that this difference can result in management failing to act to improve safety conditions, and as a result that more weight should be placed on the employee than management perspective when making program decisions. Furthermore, TWH researchers argue for a participatory approach to program development and management [58]. Thus, if small business leaders are primarily focused on TWH in a business context, as found in the present study, there are likely substantial gaps between TWH programs and employee needs and interests.

In practice, these finding suggests that small business leaders may be more receptive to TWH leadership practices if it is communicated through the lens of their business. To obtain leadership support for TWH, academics and practitioners should build an argument for why and how consideration of their employees perspective as well as their own perspective on TWH contributes to business operations and overall business success. 

It is worth noting that there may be some alternative explanations for the code frequencies we observed. As mentioned above, leaders rarely talked about specific leadership practices. However, this does not mean they do not display them in their daily work activities. It may mean that they do not naturally discuss them. We may have observed different results had we structured our questions differently. For example, we did not preface the interview with an explanation of what leadership is nor did we explain what TWH is. The latter may have helped leaders better understand of the aim of the interview and resulted in more description of their leadership practices. The former may have contributed to our finding that small business leaders primarily discussed health, safety, and well-being separately. Leaders may have interpreted the questions as asking for non-integrative responses.

### 4.1. Future Research

The present study adds to the literature on leadership support for TWH business practices [59] by being the first to begin to understand what leadership support means from small business leaders’ own words. This follows previous calls by leadership researchers for more mixed methods leadership research to better understand leadership in context [52]. Building upon the present study, future qualitative research should consider focusing interview questions on TWH as an integrative concept to better understand the ways leadership practices are used to simultaneously influence health, safety, and well-being. Additionally, concrete questions should be used to hone in on specific leadership practices such as coaching or ethics. An important next step in this research will also be to study small business TWH leadership from the employee perspective to learn whether they observe their leaders engaging in leadership practices. As described above, employee and management perceptions often differ.

Next, a quantitative needs assessment that employs a larger sample of small business leaders is needed to quantify opportunities for TWH leadership development. This should investigate which leadership styles, or combination of styles, elicit the best health, safety, and well-being outcomes including organizational-level indicators of TWH [60] as well as employee-level health, safety, and well-being outcomes. It will be important to assess TWH leadership from multiple perspectives, such as management, health and safety manager, human resources manager, and employee. Finally, future research on this topic should consider how contextual factors including business size, industry, business structure, geographic location, ownership, and other factors are associated with leadership. Other factors, including leaders’ age, gender, race, ethnicity, and identifying workforce information (e.g., diversity, industry sector, part-time, full-time) should be considered as well. 

Another next step is to investigate TWH leadership development strategies. Leadership development research for health promotion and safety has historically been siloed. Thus, an important next step will be to investigate the ways in which leadership development can be a means of improving TWH. We are currently investigating a TWH leadership development program described in Schwatka et al. [7] by structuring the learning content based on the three themes learned in this qualitative study: their business’s TWH policies and practices, their employee’s perspective of these policies and practices, and their own perspective via key leadership practices curated from multiple leadership theories. Our aim is to help small business leaders place their business practices in the context of their employees TWH needs as well as their own leadership practices.

### 4.2. Limitations

The present study had a few limitations. First, small business leaders who agreed to be interviewed may represent leaders who are more interested in TWH than all small business leaders. Results of the present study also reflect the small business leader perspectives of only 18 leaders from two states. Leaders from other states may approach health, safety and well-being differently. However, our sample was diverse in terms of industry and gender, which may strengthen the generalizability of our findings. The study findings would be strengthened if we had also interviewed small business employees. Finally, due to the way the questions were worded, we cannot say for sure whether leaders’ responses reflect the TWH integrated framework or whether they were reflecting upon what they do for health, safety, and well-being separately.

## 5. Conclusions

Leadership is a critical to TWH system effectiveness in small business. In this qualitative study we aimed to integrate both health promoting leadership and safety leadership to begin to understand small business leadership practices that protect and promote employee health. The interviews demonstrate that small business leaders care about the health of their employees, but because of the perceived value to their business, not to employees or themselves. Thus, they may lack the knowledge and skills to be successful TWH leaders. The present study supports a need for continued TWH leadership research in a small business context, including mixed methods research to understand and quantify TWH leadership practices from the small business leader and employee perspectives, as well as the development and evaluation of TWH leadership development strategies. 

## Figures and Tables

**Table 1:** Percent of child codes mentioned in leader interviews by parent code.

Parent Code	Child Code	Description	Percent of Total Parent Code
**Business**	Health and safety programs	Program in general or specific program components, such as incentives, biometrics or training	38%
	Organizational barriers	Business barriers that hinder success of health and safety program, such as multi-site work environments	10%
	Employee feedback	Systematic efforts to collect employee feedback on the health and safety program, such as during an annual review	9%
	Health communication	Communicating the importance of health in general and the health and safety program specifically via different channels, such as email	9%
	Program evaluation	Efforts to evaluate their health and safety program and adjust as needed, such as tracking flu shot uptake during a campaign to get employees to take their flu shot	7%
**Self**	Lead by example	Talking and acting in ways that are consistent with their health and safety program, such as modeling good work/life balance	25%
	Individual consideration	Efforts to personally attend to individual employee’s needs, such as regular one-on-one check-ins	20%
	Helpful strategies	Mention of a specific thing they say or do that they have found to be particularly helpful	12%
	Outcome	Perceived outcome of personally being involved in the health and safety programs, such as a better relationship with their employees	8%
	Health value	Personal value for health, safety, and well-being	6%
**Employee**	Employee barrier	Employee-specific barriers that hinder success of health and safety program, such as employees already having too much to do	26%
	Family	Recognition that employees have a family outside of work, family participation in health and safety program, or employees taking health and safety program home to their family	20%
	Employee leadership	Ways in which employees demonstrate leadership in the health and safety program, such as employees identifying hazards and working to control them	17%
	Program participation	Mention of a percent engagement in the health and safety program or ways in which employees participate	15%
	Personal accountability	Employees needing to take care of themselves and leaving health decisions ultimately up to the employee	6%

## Appendix A. Small Business Leader Interview Questions

### Appendix A.1. Organizational Mission and Vision

1. Why does health, safety, and well-being matter to you? For both your own health, safety, and well-being but also for your employees. How did you come to this understanding?
2. What does employee health, safety, and well-being look like today in your organization?
3. Now that we’ve talked about what employee health, safety, and well-being looks like today, how do you determine what the future of employee health, safety, and well-being looks like in your organization?

Follow-up Questions

How do you communicate your vision for employee health, safety, and well-being?

Do you spend time talking to your employees about health and safety in your organization?—Can you give me an example of when you did this?

### Appendix A.2. Organizational Culture

4. Every organization has its own unique culture of health and safety that develops over time based on what’s said and what’s done. What does your organization do to set the culture for healthy work? How do you involve employees?
5. Do you find it challenging, or difficult to engage your employees around health, safety, and wellness?—How do you approach this?

Follow-up Question

How do you encourage your employees to provide feedback for health and wellness in your organization?

When one of your employees provides feedback on health and wellness policies or programs, how do you react?

Have you ever incorporated employee suggestions into policies or standards? How did you do this?

Can you give me an example of how you have inspired your employees or how your employees have inspired each other to be healthier and safer in the workplace?

### Appendix A.3. Lead Yourself

6. How do you lead by example in your organization when it comes to health, safety, and well-being?
7. How do you think your actions affect the behaviors of employees? Can you provide an example?

Follow-up Question

Do you ask for feedback on how you’re leading by example from your employees? If so, how do you do it?

Employee Advancement

8. How do you go about identifying opportunities for supporting the values and goals of your employees?
9. Can you describe the opportunities and challenges you have experienced when initiating changes to improve employee health, safety and well-being?

### Appendix A.4. Feedback and Recognition

10. What methods do you use to hold yourself and your employees accountable for sustaining good health, safety and wellness practices?
11. What are some ways that you recognize your employees for prioritizing health, safety, and wellness? For example, do you use rewards, praise, celebrate?

Follow-up Questions

How do you provide feedback, whether it’s good or needs improvement, to your employees when it comes to health, safety, and wellness in your organization?

How do you solicit and receive feedback from other leaders and employees about prioritizing health, safety, and well-being in your organization?

Have you ever encountered opposition to change around employee health, safety, and well-being? How did you deal with it?

## References

[1] NIOSH (2018). What Is Total Worker Health?. https://www.cdc.gov/niosh/twh/default.html.

[2] McLellan D., Moore W., Nagler E., Sorensen G., Harvard T.H. (2017). Implementing an Integrated Approach: Weaving Employee Health, Safety, and Well-Being into the Fabric of Your Organization.

[3] Sorensen G., McLellan D., Dennerlein J.T., Pronk N.P., Allen J.D., Boden L.I., Okechukwu C.A., Hashimoto D., Stoddard A., Wagner G.R. (2013). Integration of health protection and health promotion rationale, indicators, and metrics. J. Occup. Environ. Med..

[4] Sorensen G., McLellan D., Sabbath E., Dennerlein J., Nagler E., Hurtado D., Pronk N., Wagner G. (2016). Integrating worksite health protection and health promotion: A conceptual model for intervention and research. Prev. Med..

[5] Kelloway E.K., Barling J. (2010). Leadership development as an intervention in occupational health psychology. Work Stress.

[6] McCoy K., Stinson K., Scott K., Tenney L., Newman L.S. (2014). Health promotion in small business: A systematic review of factors influencing adoption and effectiveness of worksite wellness programs. J. Occup. Environ. Med..

[7] Schwatka N., Tenney L., Dally M., Scott J., Brown C., Weitzenkamp D., Shore E., Newman L. (2018). Small business total worker health: A conceptual and methodological approach to facilitating organizational change. Occup. Health Sci..

[8] Cunningham T.R., Sinclair R., Schulte P. (2014). Better understanding the small business construct to advance research on delivering workplace health and safety. Small Enterp. Res..

[9] Dinh J.E., Lord R.G., Gardner W.L., Meuser J.D., Liden R.C., Hu J. (2014). Leadership theory and research in the new millennium: Current theoretical trends and changing perspectives. Leadersh. Q..

[10] Alvesson M. (1989). The culture perspective on organizations: Instrumental values and basic features of culture. Scand. J. Manag..

[11] Kouzes J.M., Posner B.Z. (2012). The Leadership Challenge: How to MAke Extraordinary Things Happen in Organizations.

[12] Hogan R., Kaiser R.B. (2005). What we know about leadership. Rev. Gen. Psychol..

[13] Allen G.P., Moore W.M., Moser L.R., Neill K.K., Sambamoorthi U., Bell H.S. (2016). The role of servant leadership and transformational leadership in academic pharmacy. Am. J. Pharm. Educ..

[14] Graen G.B., Uhl-Bien M. (1995). Relationship-based approach to leadership: Development of leader-member exchange (lmx) theory of leadership over 25 years: Applying a multi-level multi-domain perspective. Leadersh. Q..

[15] Sirota D., Mischkind L., Meltzer M. (2005). The Enthusiastic Employee: How Companies Profit by Giving Workers What They Want.

[16] Arnold K.A., Turner N., Barling J., Kelloway E.K., McKee M.C. (2007). Transformational leadership and psychological well-being: The mediating role of meaningful work. J. Occup. Health Psychol..

[17] Sosik J., Godshalk V. (2000). Leadership styles, mentoring functions received, and job-related stress: A conceptual model and preliminary study. J. Organ. Behav..

[18] Munir F., Nielsen K., Carneiro I.G. (2010). Transformational leadership and depressive symptoms: A prospective study. J. Affect. Disord..

[19] Munir F., Nielsen K. (2009). Does self-efficacy mediate the relationship between transformational leadership behaviours and healthcare workers’ sleep quality? A longitudinal study. J. Adv. Nurs..

[20] Clarke S. (2013). Safety leadership: A meta-analytic review of transformational and transactional leadership styles as antecedents of safety behaviours. J. Occup. Organ. Psychol..

[21] Nahrgang J.D., Morgeson F.P., Hofmann D.A. (2011). Safety at work: A meta-analytic investigation of the link between job demands, job resources, burnout, engagement, and safety outcomes. J. Appl. Psychol..

[22] Christian M.S., Bradley J.C., Wallace J.C., Burke M.J. (2009). Workplace safety: A meta-analysis of the roles of person and situation factors. J. Appl. Psychol..

[23] Nyberg A., Alfredsson L., Theorell T., Westerlund H., Vahtera J., Kivimaki M. (2009). Managerial leadership and ischaemic heart disease among employees: The swedish wolf study. Occup. Environ. Med..

[24] Karlin W., Brondolo E., Schwartz J. (2003). Workplace social support and ambulatory cardiovascular activity in new york city traffic agents. Psychosom. Med..

[25] Bamberger P.A., Bacharach S.B. (2006). Abusive supervision and subordinate problem drinking: Taking resistance, stress, and subordinate personality into account. Hum. Relat..

[26] Erikson W. (2005). Work factors as predictors of smoking relapse in nurses’ aides. Int. Arch. Occup. Environ. Health.

[27] Skakon J., Nielsen K., Borg V., Guzman J. (2010). Are leaders' well-being, behaviours and style associated with the affective well-being of their employees? A systematic review of three decades of research. Work Stress.

[28] Colquitt J.A., Conlon D.E., Wesson M.J., Porter C.O.L.H., Ng K.Y. (2001). Justice at the milleninium: A meta-analytic review of 25 years of organizational justice research. J. Appl. Psychol..

[29] Nielsen K., Randall R., Yarker J., Brenner S.-O. (2008). The effects of transformational leadership on followers’ perceived work characteristics and psychological well-being: A longitudinal study. Work Stress.

[30] Eriksson A., Axelsson R., Bihari Axelsson S. (2011). Health promoting leadership—Different views of the concept. Work J. Prev. Assess. Rehabil..

[31] Skarholt K., Bli E., Sandsund M., Andersen T. (2016). Health promoting leadership practices in four norweigan industries. Health Promot. Int..

[32] Jiménez P., Winkler B., Dunkl A. (2016). Creating a healthy working environment with leadership: The concept of health-promoting leadership. Int. J. Hum. Resour. Manag..

[33] Winkler E., Busch C., Clasen J., Vowinkel J. (2014). Leaderhsip behavior as a health-promoting resoruces for workers in low-skillsed jobs and the moderating role of power distance orientation. Ger. J. Hum. Resour. Manag..

[34] Dunkl A., Jimenez P., Sarotar Zizek S., Milfeiner B., Kallus W.K. (2015). Similarities and differences of health-promoting leadership and transformational leadership. Our Econ..

[35] Hofmann D.A., Morgeson F.P., Barling J., Frone M. (2004). The Role of Leadership in Safety.

[36] Bass B. (1985). Leadership and Performance Beyond Expectations.

[37] Barling J., Loughlin C., Kelloway E.K. (2002). Development and test of a model linking safety-specific transformational leadership and occupational safety. J. Appl. Psychol..

[38] Kelloway E., Mullen J., Francis L. (2006). Divergent effects of transformational and passive leadership on employee safety. J. Occup. Health Psychol..

[39] Hoffmeister K., Gibbons A.M., Johnson S.K., Cigularov K.P., Chen P.Y., Rosecrance J.C. (2014). The differential effects of transformational leadership facets on employee safety. Saf. Sci..

[40] Mullen J., Kelloway E. (2009). Safety leadership: A longitudinal study of the effects of transformational leadership on safety outcomes. J. Occup. Organ. Psychol..

[41] von Thiele Schwarz U., Augustsson H., Hasson H., Stenfors-Hayes T. (2015). Promoting employee health by integrating health protection, health promotion, and continuous improvement. J. Occup. Environ. Med..

[42] Hofmann D., Morgeson F., Gerras S. (2003). Climate as a moderator of the relationship between leader-member exchange and content specific citizenship: Safety climate as an exemplar. J. Appl. Psychol..

[43] Martínez-Córcoles M., Schöbel M., Gracia F.J., Tomás I., Peiró J.M. (2012). Linking empowering leadership to safety participation in nuclear power plants: A structural equation model. J. Saf. Res..

[44] McLellan D.L., Williams J.A., Katz J.N., Pronk N.P., Wagner G.R., Caban-Martinez A.J., Nelson C.C., Sorensen G. (2017). Key organizational characteristics for integrated approaches to protect and promote worker health in smaller enterprises. J. Occup. Environ. Med.

[45] Avolio B.J., Gardner W.L. (2005). Authentic leadership development: Getting to the root of positive forms of leadership. Leadersh. Q..

[46] Uhl-Bien M. (2006). Relational leadership theory: Exploring the social processes of leadership and organizing. Leadersh. Q..

[47] van Dierendonck D. (2011). Servant leadership: A review and synthesis. J. Manag..

[48] (2016). Dedoose.

[49] Saldana J. (2012). The coding Manual for Qualitative Researchers.

[50] Bolden R. (2011). Distributed leadership in organizations: A review of theory and research. Int. J. Manag. Rev..

[51] Brown M.E., Treviño L.K. (2006). Ethical leadership: A review and future directions. Leadersh. Q..

[52] Avolio B.J., Walumbwa F.O., Weber T.J. (2009). Leadership: Current theories, research, and future directions. Annu. Rev. Psychol..

[53] Huang Y.H., Leamon T.B., Courtney T.K., Chen P.Y., DeArmond S. (2007). Corporate financial decision-makers: Perceptions of workplace safety. Accid. Anal. Prev..

[54] Pescud M., Teal R., Shilton T., Slevin T., Ledger M., Waterworth P., Rosenberg M. (2015). Employers’ views on the promotion of workplace health and wellbeing: A qualitative study. BMC Public Health.

[55] Burke W., Litwin G. (1992). A causal model of organizational performance. J. Manag..

[56] Huang Y.-H., Robertson M.M., Lee J., Rineer J., Murphy L.A., Garabet A., Dainoff M.J. (2014). Supervisory interpretation of safety climate versus employee safety climate perception: Association with safety behavior and outcomes for lone workers. Transp. Res. Part F Traffic Psychol. Behav..

[57] Gittleman J.L., Gardner P.C., Haile E., Sampson J.M., Cigularov K.P., Ermann E.D., Stafford P., Chen P.Y. (2010). Citycenter and cosmopolitan construction projects, las vegas, nevada: Lessons learned from the use of multiple sources and mixed methods in a safety needs assessment. J. Saf. Res..

[58] Punnett L., Warren N., Henning R., Nobrega S., Cherniack M., CPH-NEW Research Team (2013). Participatory ergonomics as a model for integrated programs to prevent chronic disease. J. Occup. Environ. Med..

[59] McLellan D.L., Caban-Martinez A.J., Nelson C.C., Pronk N.P., Katz J.N., Allen J.D., Davis K.L., Wagner G.R., Sorensen G. (2015). Organizational characteristics influence implementation of worksite health protection and promotion programs. J. Occup. Environ. Med..

[60] Williams J.A.R., Nelson C.C., Caban-Martinez A.J., Katz J.N., Wagner G.R., Pronk N.P., Sorensen G., McLellan D.L. (2015). Validation of a new metric for assessing the integration of health protection and health promotion in a sample of small- and medium-sized employer groups. J. Occup. Environ. Med..

